# mRNA Subtype of Cancer-Associated Fibroblasts Significantly Affects Key Characteristics of Head and Neck Cancer Cells

**DOI:** 10.3390/cancers14092286

**Published:** 2022-05-03

**Authors:** Barbora Peltanová, Hana Holcová Polanská, Martina Raudenská, Jan Balvan, Jiří Navrátil, Tomáš Vičar, Jaromír Gumulec, Barbora Čechová, Martin Kräter, Jochen Guck, David Kalfeřt, Marek Grega, Jan Plzák, Jan Betka, Michal Masařík

**Affiliations:** 1 Department of Pathological Physiology, Faculty of Medicine, Masaryk University, Kamenice 5, 62500 Brno, Czech Republic; bpeltanova@seznam.cz (B.P.); hana.polanska@gmail.com (H.H.P.); m.raudenska@gmail.com (M.R.); jan.balvan@med.muni.cz (J.B.); jiri.navratil@med.muni.cz (J.N.); j.gumulec@gmail.com (J.G.); cechova.b97@gmail.com (B.Č.); 2 Department of Physiology, Faculty of Medicine, Masaryk University, Kamenice 5, 62500 Brno, Czech Republic; tomasvicar@gmail.com; 3 Department of Chemistry and Biochemistry, Mendel University in Brno, Zemedelska 1, 61300 Brno, Czech Republic; 4 Max Planck Institute for the Science of Light, Staudtstraße 2, 91058 Erlangen, Germany; martin.kraeter@mpl.mpg.de (M.K.); jochen.guck@mpl.mpg.de (J.G.); 5 Department of Otorhinolaryngology and Head and Neck Surgery, First Faculty of Medicine, University Hospital Motol, Charles University, V Uvalu 84, 15006 Prague, Czech Republic; david.kalfert@fnmotol.cz (D.K.); jan.plzak@fnmotol.cz (J.P.); jan.betka@fnmotol.cz (J.B.); 6 Department of Pathology and Molecular Medicine, 2nd Faculty of Medicine, University Hospital Motol, Charles University, V Uvalu 84, 15006 Prague, Czech Republic; marek.grega@fnmotol.cz; 7 BIOCEV, First Faculty of Medicine, Charles University, Prumyslova 595, 25250 Vestec, Czech Republic

**Keywords:** cancer, HNSCC, cancer-associated fibroblasts, tumour microenvironment, cell stiffness

## Abstract

**Simple Summary:**

The interplay between cancer-associated fibroblasts (CAFs) and head and neck cancer cells and its effect on the metabolic state, mechanobiology, and aggressiveness of cancer cells is described. In vitro coculture techniques were used. Not all CAFs support tumours–the right kind of cooperation between cancer cells and CAFs is needed for tumour growth and progression, and only specific mRNA subtypes of CAFs can support the growth of primary cancer cells or metastases carrying different mutational statuses.

**Abstract:**

Head and neck squamous cell carcinomas (HNSCC) belong among severe and highly complex malignant diseases showing a high level of heterogeneity and consequently also a variance in therapeutic response, regardless of clinical stage. Our study implies that the progression of HNSCC may be supported by cancer-associated fibroblasts (CAFs) in the tumour microenvironment (TME) and the heterogeneity of this disease may lie in the level of cooperation between CAFs and epithelial cancer cells, as communication between CAFs and epithelial cancer cells seems to be a key factor for the sustained growth of the tumour mass. In this study, we investigated how CAFs derived from tumours of different mRNA subtypes influence the proliferation of cancer cells and their metabolic and biomechanical reprogramming. We also investigated the clinicopathological significance of the expression of these metabolism-related genes in tissue samples of HNSCC patients to identify a possible gene signature typical for HNSCC progression. We found that the right kind of cooperation between cancer cells and CAFs is needed for tumour growth and progression, and only specific mRNA subtypes can support the growth of primary cancer cells or metastases. Specifically, during coculture, cancer cell colony supporting effect and effect of CAFs on cell stiffness of cancer cells are driven by the mRNA subtype of the tumour from which the CAFs are derived. The degree of colony-forming support is reflected in cancer cell glycolysis levels and lactate shuttle-related transporters.

## 1. Introduction

Head and neck squamous cell carcinoma (HNSCC) is the sixth most prevalent cancer worldwide [1] and represents a heterogeneous group of tumours originating from mucosal epithelial cells that line the nasal and oral cavity, oropharynx, hypopharynx, and larynx. In recent years, it has been shown that cells within the tumour stroma drastically modulate the behaviour of tumour cells [2]. The predominant cell populations within the tumour microenvironment (TME) are cancer-associated fibroblasts (CAFs). In HNSCC, the late stages of carcinomas frequently contain about 80% of CAFs [3].

CAFs were originally presumed to represent a homogeneous group of stromal cells whose main role was to provide and maintain a favourable TME to promote the growth, survival, and metastatic spread of tumour cells. However, CAFs display a great heterogeneity regarding their actual tumour-promoting or tumour-inhibiting properties. The wide range and heterogeneity of functions attributed to CAFs raise the question of whether this cell population executes all these functions or whether specific subpopulations of CAFs perform specific tasks, such as supporting proliferation or promoting metastatic potential. This can also implicate possible overlaps of different CAF subpopulations or a possible switch between different functional states. It is crucial to point out that solid tumours display, not only histological and morphological heterogeneity, but also intertumoral, metabolic heterogeneity and metabolic plasticity. This characteristic is not restricted only to cancer cells, but it can also be found in surrounding stromal cells [4,5].

It has been observed that cancer cells and CAFs can establish a metabolic symbiosis in which cancer cells corrupt normal fibroblasts, which in turn produce metabolites for cancer cells to supply their metabolic needs. In addition to glucose, cancer cells can also utilize lactate as an alternative energy source via lactate shuttle, which is regulated by the conversion of lactate into pyruvate, as well as by the transport of lactate across the cancer cell plasma membrane. Interestingly, aerobic glycolysis can also take place in CAFs rather than in epithelial cells, which then, in turn, supplies cancer cells with lactate for OXPHOS. This lactate exchange between cancer cells and CAFs is a process known as the reverse Warburg effect [6]. Nevertheless, metabolic symbiosis may also occur between oxidative CAFs and glycolytic tumour cells, which has also been observed in HNSCC [5].

Apart from metabolic symbiosis, CAFs are known to support cancer cell migration [7], invasiveness, and metastasis formation [8]. Cell mechanic phenotypes largely impact the cell’s ability to migrate and invade due to changes in the cytoskeleton. Accordingly, the epithelial–mesenchymal transition (EMT) is accompanied by significant changes in cell stiffness [9]. Processes enabling migration are energetically demanding, and these demands further increase with increasing cell stiffness [10].

In our study, the classification to the mRNA cluster was based on Lawrence et al. [11] and modified accordingly: Basal phenotype was classified by high *TP63* and *EGFR* and low *SOX2* expression. The mesenchymal phenotype was classified by high *VIM*, *DES*, and low *TP63* expression, the classical phenotype by high expression of *SOX2*, *NFE2L2*, and positive smoking history, the atypical phenotype was characterised by high *SOX2* expression and p16 positivity. The mean of standardised log2 expression/smoking/p16 status values was calculated, and the patient was attributed to the category with the highest mean value (based on Lawrence et al. [11]). In this study, we investigated how CAFs derived from tumour tissues of different mRNA subtypes influence the proliferation, metabolic, and biomechanical reprogramming of cancer cells. We also investigated the clinicopathological significance of the expression of these metabolism-related genes in tissue samples of HNSCC patients to identify a possible gene signature typical for HNSCC progression. We assume that the right kind of cooperation between cancer cells and CAFs is needed for tumour growth and progression.

## 2. Material and Methods

### 2.1. Tumour Samples Collection

The study was approved by the ethical committee of Motol University Hospital, Prague, Czech Republic. All surgical tissue samples were obtained from HNSCC patients after they signed the informed consent documents. Patients were completely clinically examined, and tumour samples were taken from verified HNSCC under general anaesthesia; the inclusion criteria were as follows: histologically confirmed squamous cell carcinoma and no previous oncologic treatment; each tissue specimen underwent pathology quality control. Hematoxylin and eosin-stained sections from each sample were subjected to independent pathology review to confirm squamous cell carcinoma histological consistence. Tumour samples with approximately >60% tumour nuclei and <20% necrosis were considered sufficient for the following analyses. The tissue material harvested during surgery was divided into two pieces, the first was stored in an RNAlater (Ambion, Austin, TX, USA) for later RNA analyses. The second portion of the sample was placed into the cultivation medium (MEM) with the addition of 1% trypsin and 1% antibiotic-antimycotic solution (Santa Cruz Biotechnology, Dallas, TX, USA). The material was maintained at cold temperatures for 16–24 h to enable trypsin diffusion. Primary cell lines were subsequently prepared.

### 2.2. Ex-Vivo Cell Cultures

The tissue material was first rinsed with 50% ethanol (Merck, Darmstadt, Germany), followed by phosphate buffer (Invitrogen, Waltham, MA, USA). The necrotic and adipose tissues were then removed from the tissue using a sterile scalpel, and the tumour tissue was cut into 3 mm pieces. Next, samples in the trypsin media were kept at 37 °C to activate the trypsin. Subsequently, the tumour pieces were transferred to a sterile tube with phosphate buffer and centrifuged at 4 °C, 2700 rpm for 7 min. After removing the supernatant, the tumour pieces were placed into the culture medium (MEM) with added 10% FBS (Biochrom, Holliston, MA, USA), 1% antibiotic-antimycotic solution, 10 μg/mL gentamicin sulfate, and 10 μg/mL ciprofloxacin (Santa Cruz Biotechnology, Dallas, TX, USA) to prevent contamination. After 7 days the culture medium was replaced with MEM medium with 10% FBS, supplemented with antibiotics (penicillin 100 U/mL and streptomycin 0.1 mg/mL) (Biochrom, Holliston, MA, USA) and 0.4 μg/mL hydrocortisone (Merck, Darmstadt, Germany). Since we were primarily interested in the effect of TME-derived fibroblasts (CAFs), selection using population overgrowth was performed. The passages of CAFs ranged from 1 to 5.

### 2.3. Cell Lines

Two human head and neck squamous cell carcinoma cell lines were used in this study. FaDu (RRID:CVCL_1218) is an epithelial adherent cell line derived from hypopharyngeal carcinoma. Detroit 562 (RRID:CVCL_1171) is an epithelial adherent cell line derived from the pleural effusion of metastatic pharyngeal carcinoma. Authenticated cell lines were purchased from ATCC (Manassas, VA, USA) within the last three years.

### 2.4. Cell Lines Cultivation

FaDu and Detroit 562 cells were cultured in MEM medium with 10% FBS, supplemented with antibiotics (penicillin 100 U/mL and streptomycin 0.1 mg/mL) and 0.4 μg/mL hydrocortisone. Cells were maintained at 37 °C in a humidified incubator with 5% CO_2_ (Sanyo, Moriguchi, Osaka, Japan). The passages of all cell lines performed in our lab ranged from 5 to 10. All experiments were performed with mycoplasma-free cells. Mycoplasma was detected by PCR (primers MYCO_A GGCGAATGGGTGAGTAACACG and MYCO_B CGGATAACGCTTGCGACCTATG).

### 2.5. Conditioned Media Preparation

Cell-conditioned media were collected from 90% confluent FaDu, Detroit 562 cell lines, and the CAFs were cultivated for 24 h. The collected media were frozen at −20 °C until further use.

### 2.6. Colony-Forming Assay

The FaDu and Detroit 562 cell lines were cocultured with the CAFs using the Corning^®^ Transwell^®^ cell culture inserts (Merck, Darmstadt, Germany). The FaDu and Detroit 562 cells were seeded in 6-well plates, while the CAFs were seeded in inserts and left to adhere overnight. The seeding density was 100 cancer cells in the bottom chamber and 5000 CAFs in the insert (1:50 ratio) to form colonies in 3 weeks. After cultivation, colonies were fixed with cold methanol, visualised by trypan blue and analysed. For analysis, regions of interest were chosen by registration of each image to the reference image (with the manually labelled area of interest). Next, the colonies were segmented by thresholding of the blue component of the image, where a single fixed threshold was used. Finally, the fraction of areas covered by colonies was computed.

### 2.7. RNA Isolation and Reverse Transcription

The FaDu and Detroit 562 cell lines were cocultured with the CAFs using Transwell^®^ cell culture inserts. The FaDu and Detroit 562 cells were seeded in 6-well plates at a density of 2 × 10^4^, while the CAFs were seeded at a density of 5 × 10^3^ in inserts and left to adhere overnight. The inserts were then inserted into the wells and cocultures were cultured for 7 days. After 7 days, the medium was collected, and cells were harvested for RNA isolation. TriPure Isolation Reagent (Roche, Bazel, Switzerland) was used for RNA isolation from tissue and harvested cells. The isolated RNA was used for cDNA synthesis. The RNA (1000 ng) was transcribed using the High-Capacity cDNA Reverse Transcription Kit (Applied Biosystems, Waltham, MA, USA), which was used according to the manufacturer’s instructions. cDNA (20 μL) prepared from total RNA was diluted with RNase-free water to 100 μL, and the amount of 5 μL was directly analysed.

### 2.8. Quantitative Real-Time Polymerase Chain Reaction

qRT-PCR was performed using TaqMan gene expression assays with the LightCycler^®^480 II System (Roche, Bazel, Switzerland) and the amplified cDNA was analysed by the comparative Ct method using PSMB2 as a housekeeping gene. Primer and probe sets for PSMB2 (Hs01009704_m1), SOX2 (Hs01053049_s1), EGFR (Hs01076078_m1), TP63 (Hs00978340_m1), VIM (Hs00958111_m1), DES (Hs00157258_m1), NFE2L2 (Hs00975961_g1), ACTA2 (Hs05005341_m1), MCT1 (Hs00161826_m1), MCT4 (Hs00358829_m1), CD147 (Hs00174305_m1), CAV1 (Hs00971716_m1), were selected from the TaqMan gene expression assays (Thermo Fisher Scientific, Waltham, MA, USA). qRT-PCR was performed under the following amplification conditions: total volume 20 μL, initial incubation at 50 °C/2 min followed by denaturation at 95 °C/10 min, then 45 cycles at 95 °C/15 s and 60 °C/1 min.

### 2.9. Lactate Assay

L-lactate levels in coculture media were quantitatively measured with a colourimetric L-lactate assay kit (Abcam, Cambridge, UK) according to the manufacturer’s protocol. Briefly, coculture media and reaction mix, consisting of assay buffer, substrate mix, and enzyme mix were added into a 96-well plate. After incubation for 30 min at room temperature, the colour signal was measured at 450 nm.

### 2.10. Live-Cell Metabolic Assay

The assay was performed according to the manufacturer’s instructions. Briefly, to examine the effect of the HNSCC CAF-derived conditioned media, the FaDu and Detroit 562 cells were seeded at the density of 1 × 10^4^ cells per well in a Seahorse 8-well plate (Agilent Technologies, Santa Clara, CA, USA) and incubated for 24 h at 37 °C in the CAF-derived conditioned media. A day before the analysis, Seahorse cartridge (Agilent Technologies) was hydrated and incubated at 37 °C without CO_2_. On the day of the experiment, the Seahorse XFp analyzer (Agilent Technologies, Santa Clara, CA, USA) was calibrated using hydrated cartilage. The culture medium was changed to Seahorse XF RPMI medium, pH 7.4 (Agilent Technologies, Santa Clara, CA, USA). After incubating cells for 1 h at 37 °C without CO_2_, the Seahorse assay was performed by measuring the extracellular acidification rates (ECAR) as a proxy for glycolytic readouts and the oxygen consumption rates (OCR) as a proxy for oxidative phosphorylation readouts. The data were acquired and analysed using Wave Controller 2.4 software (Agilent Technologies, Santa Clara, CA, USA).

### 2.11. Immunohistochemistry

Representative FFPE tumour tissue blocks were selected for immunohistochemical analysis. Immunohistochemistry was performed on 2 µm sections using specific primary antibodies against p16 (BD Pharmingen™, BD Biosciences, Franklin Lakes, NJ, USA, clone G175-405, dilution 1:100), MCT1 (Abcam, Cambridge, UK, clone SLC16A1, ab90582, 1:100), MCT4 (Merck, Darmstadt, Germany, clone SLC16A3, HPA021451, 1:300), CD147 (Abcam, Cambridge, UK, clone ab666, 1:100), IRF3 (Abcam, Cambridge, UK, clone ab68481, 1:500) and α-SMA (Abcam, Cambridge, UK, clone ab5694, 1:300). Antigen demasking was performed at 99 °C, by pH6 or pH9. As a secondary antibody, a Mouse/Rabbit PolyDetector HRP Label (BioSB, Goleta, CA, USA) was used. All the procedures were performed according to added datasheets.

We assessed the expressions as negative or positive. In positive cases, we graded the intensity into three grades (1+ weak staining; 2+ moderate; 3+ strong positivity). At staining of p16, we evaluated the intensity, percentage of positive cells, and location of expression (nuclear and/or cytoplasmic) in tumour cells. The expressions of the remaining antibodies were assessed in tumour cells, and in peritumoral tissue (inflammatory cells, stromal elements), evaluating intensity, location of expression (cytoplasm, membrane), and coarse estimating the number of positive cells.

### 2.12. Atomic Force Microscopy

For the analysis of cell stiffness of FaDu and Detroit 562 cells, the cells were cultured in the CAF-derived conditioned media for 24 h. Then, the bioAFM microscope JPK NanoWizard 3 (JPK BioAFM, Berlin, Germany) was placed on the inverted optical microscope Olympus IX-81 (Olympus, Tokyo, Japan) and equipped with a fluorescence and confocal module, thus allowing a combined experiment (AFM-optical combined images). The maximal scanning range of the AFM microscope in the X-Y-Z range was 100-100-15 µm. The typical approach/retract settings were identical with a 15 μm extend/retract length, a setpoint value of 1 nN, a pixel rate of 2048 Hz, and a speed of 30 µm/s. The system operated under closed-loop control. After reaching the selected contact force, the cantilever was retracted. The retraction length of 15 μm was sufficient to overcome any adhesion between the tip and the sample, and to make sure that the cantilever had been completely retracted from the sample surface. A force-distance (FD) curve was recorded at each point of the cantilever approach/retract movement. AFM measurements were obtained at 37 °C with force measurements recorded at a pulling speed of 30 µm/s (extension time 0.5 s).

The Young’s modulus (E) was calculated by fitting the Hertzian–Sneddon model on the FD curves measured as force maps (64 × 64 points) of the region containing either a single cell or multiple cells. JPK data evaluation software was used for the batch processing of measured data. The adjustment of the cantilever position above the sample was carried out under the microscope by controlling the position of the AFM-head by a motorised stage, equipped with a Petri dish heater (JPK) allowing a precise positioning of the sample together with a constant elevated temperature of the sample for the whole period of the experiment. Soft uncoated AFM probes HYDRA-2R-100N (Applied NanoStructures, Mountain View, CA, USA), i.e., silicon nitride cantilevers with silicon tips, are used for stiffness studies because they are maximally gentle to living cells (not causing mechanical stimulation). Moreover, as compared with coated cantilevers, these probes are very stable under elevated temperatures in liquids—thus allowing long-time measurements without nonspecific changes in the measured signal.

### 2.13. Quantitative Phase Imaging

The migration capacity of the FaDu cells before and after coculture with conditioned media was assessed using Tescan multimodal holographic microscope Q-PHASE. The Q-PHASE is based on the original concept of the coherence-controlled holographic microscope. Measurements were carried out under conditions optimised in our previous study [12]. Briefly, cells were cultivated in flow chambers μ-Slide I Lauer Family (Ibidi GmbH, Gräfelfing, Germany). To image enough cells in one field of view, the Nikon Plan 10/0.30 lens was chosen. Holograms were captured by a CCD camera (XIMEA, Marianka, Slovakia). Complete image reconstruction and image processing were performed in Q-PHASE control software.

### 2.14. Flow Cytometry

To verify the purity of the CAF cell culture, the cells were stained for the mesenchymal surface marker CD90 and analysed by flow cytometry. The cells were prepared as a single-cell suspension for FACS staining. For CD90 staining, cells were stained with APC-CD90 antibody (Biolegend, San Diego, CA, USA) for 30 min at 4 °C. CD90+ and CD90− cells were identified based on isotype gating. The stained cells were acquired for analysis on a FACS Aria II flow cytometer (BD Biosciences, Franklin Lakes, NJ, USA). Staining for dead cells was performed using SYTOXblue staining. Flow cytometry data were analysed with FlowJo software (BD Biosciences, Franklin Lakes, NJ, USA).

### 2.15. Real-Time Deformability Cytometry

Real-time deformability cytometry (RT-DC) was performed according to Rosendahl et al. [13] using an AcCellerator (Zellmechanik, Dresden, Germany) at 37 °C. After 48 h of indirect coculture of cancer cells with the CAF-derived media, the cells were trypsinised and kept on a roller in the culture medium in an incubator for 30 min to reduce their surface/area ratios. Subsequently, cells were transferred to the measurement buffer CellCarrier (1xPBS with 0.5% methylcellulose and adjusted to a specific viscosity) and measured using RT-DC in 30 μm wide channel microfluidic chips at a total flow of 0.240 μL/s. The acquisition was performed using ShapeIn 2.0.8 software (Zellmechanik, Dresden, Germany). The Young’s modulus was calculated from deformation and cell area using ShapeOut software [14], after filtering with the following setting: cell area 70–2000 μm^2^ (removal of cell debris/multicellular clusters), porosity 1.00–1.05 (removal of damaged cells). Measurements were performed in duplicates per treatment with a minimum of 1000 filtered cells per measurement.

### 2.16. Statistical Analysis

Pearson correlations were performed to calculate correlation coefficients between the colony-forming assay and gene expression. A one-sample *t*-test was used to compare gene expression of cocultures vs. controls. R 4.0.2 [15] was used for analysis with the following packages: ggplot2 [16], gplots 3.0.4 [17], heatmap.plus 1.3 [18], Corrplot 0.84 [19], ggpubr 0.4.0 [20]. Survival analysis was performed with Kaplan–Meier curves and using Cox proportional hazards model with Stepwise model selection (backward and forward) with the packages MASS4 [21], survival 3.2–11 [22] and survminer 0.4.9 [23]. Unless noted otherwise, *p*-level < 0.05 was considered significant.

Classification to mRNA cluster was based on [11] and modified accordingly: Basal phenotype was classified by high *TP63* and *EGFR* and low *SOX2* expression. The mesenchymal phenotype was classified by high *VIM*, *DES*, and low *TP63* expression, the classical phenotype was characterised by high expression of *SOX2* and *NFE2L2,* and positive smoking history, atypical by high *SOX2* expression and p16 positivity. The mean of standardised log2 expression/smoking/p16 status values was calculated and the patient was attributed to the category with the highest mean value.

## 3. Results

### 3.1. Clinical Characterization of Patients and Tumours Used for CAF Preparation

A total of 55 patients with head and neck squamous cell cancers were used in the study. The cohort was defined as follows: squamous cell carcinoma histology, curative therapeutic intention using surgery +/− radio or radio/chemotherapy, no distant metastatic dissemination. Tumours with the following locations were included: oropharynx (*N* = 24), larynx (*N* = 16), hypopharynx (*N* = 6), tongue (*N* = 5), the floor of the mouth (*N* = 3), and oral cavity (*N* = 1). The p16 status was analysed in all subjects. TNM8 system was used. The description of patients is shown in Table 1, Tables S1 and S6. Of those patients, a randomly selected subset of 18 patient tumour tissues was used to prepare cancer-associated fibroblast cultures (Tables S2 and S5). Its lineage specificity was confirmed, and these CAF cultures were consequently used for coculture experiments with existing cancer cell lines.

### 3.2. Lineage Specificity of Patient-Derived CAFs and Model Cancer Cells

**Figure 1:**
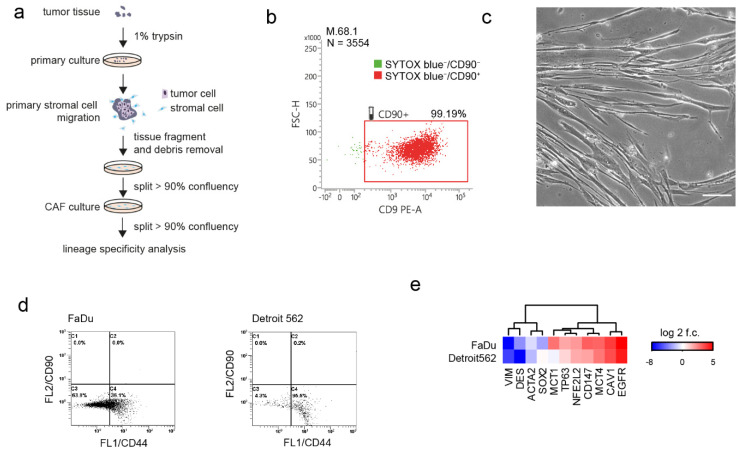
Lineage specificity of cancer-associated fibroblast (CAF) culture and cancer cells. (**a**) schematic of stromal cell isolation from tumour tissue fragments. (**b**) CD90 lineage specificity of CAF culture, positivity shown for viable = SYTOX™ Blue-negative cell subpopulation (SYTOX™ Blue stains dead cells with compromised plasma membranes but will not cross intact cell membranes), for more cases see Figure S1. (**c**) morphology of CAFs, scalebar indicate 10 μm. (**d**) CD44/CD90 status of FaDu and Detroit 562 head and neck cancer cell lines. (**e**) mRNA subtype-relevant genes, mean expression in FaDu and Detroit 562 cancer cell lines.

Based on the low doubling time of fibroblasts and their rapid growth, population overgrowth was performed. Population overgrowth of patient-derived cell cultures by CAFs was successfully reached after 2 passages (Figure 1a). Our selection method was confirmed by flow cytometry of three randomly selected early passage CAFs. The CD90 (a CAFs marker [24]) positivity of these selected cultures ranged between 79% and 99% (Figure 1b and Figure S1), indicating the successful establishment of the CD90+ CAF population. The CAFs cell cultures showed typical spindle-shaped cell morphology (Figure 1c).

FaDu is an epithelial adherent cell line derived from a primary squamous cell hypopharyngeal tumour that was chosen as the wt-PI3K model. Detroit 562 is an epithelial adherent cell line derived from pleural effusion metastasis of pharyngeal carcinoma with the PI3K activating mutation. Both cell lines are HPV negative. FaDu cells are heterozygous for TP53 missense mutation (p.R248L) and Detroit 562 cells are homozygous for missense mutation p.R175H [25]. First, the CD90/CD44 status of these model cells was verified by flow cytometry (see Figure 1d), as CD44 has been reported to be involved in tumour growth and metastasis in head and neck squamous cell cancer [26]. FaDu showed predominantly CD44/CD90 negativity, while 95.5% of Detroit 562 cells showed CD44+/CD90− status. Furthermore, the mRNA expression profile of FaDu and Detroit cells was performed. Neither FaDu nor Detroit 562 cells showed the mesenchymal phenotype characterised by high expression of EMT markers, such as vimentin (*VIM*) and desmin (*DES*). Both cell lines were characterised by high expression of *EGFR* and *NFE2L2*. FaDu cells showed low levels of the *SOX2* expression relative to the expression of *TP63*, in Detroit 562 cells the expression of *SOX2* was higher relative to the expression of *TP63.* FaDu had a higher expression of *MCT1* and *CD147* compared to Detroit 562 cells (see Figure 1e).

### 3.3. CAF-Cancer Cell Metabolic Symbiosis Is mRNA-Subtype-Specific

**Figure 2:**
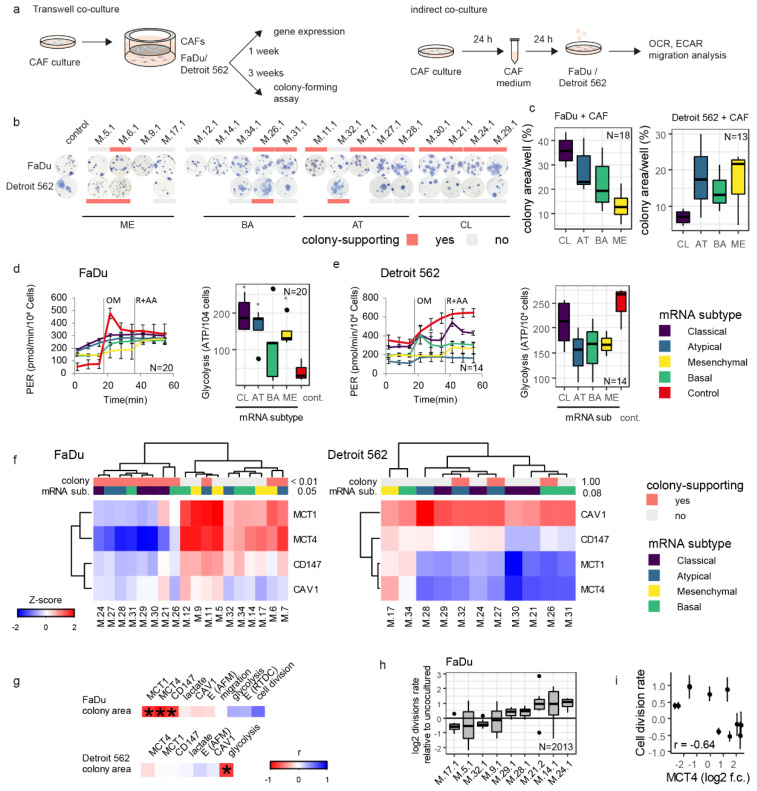
Cancer cell colony-forming capacity and lactate shuttling are reprogrammed by CAFs through coculture. (**a**) schematic of Transwell coculture and indirect coculture with conditioned media. (**b**) colony-forming assay, cancer cells Transwell-cultured with CAFs; colony-supporting indicate *p* < 0.05 for colony size vs. non-cocultured control (see Figure S2). Measurements were performed in triplicate. (**c**) cancer cell colony area affected by CAFs derived from tumour tissues of varying mRNA subtypes. CL, classical, AT, atypical, BA, basal, ME, mesenchymal. CAFs derived from tumour tissues of different mRNA subtypes affect FaDu (**d**) and Detroit 562 (**e**) glycolytic ATP production differently. Performed in triplicate; PER = Proton efflux rate, rate of protons extruded into the extracellular medium during glycolysis. (**f**) expression of lactate-shuttle-relevant genes in cancer cell lines is associated with tumour tissue-of-origin mRNA subtype and colony-supporting status after CAF coculture. (**g**) correlation heatmap of the colony-forming assay with lactate-shuttle-relevant genes, lactate in medium, cells’ Young’s modulus and ATP production from glycolysis. An asterisk indicates a statistically significant correlation at *p* < 0.05 according to the Fischer-exact test for cluster vs. colony-supporting status and mRNA subtype. (**h**) Division rate of FaDu cells cocultured with CAFs relative to non-cocultured cells (0 indicates non-cocultured). N indicate the number of divisions detected in timelapse in 74 fields of view. (**i**) cell division rate correlates negatively with MCT4 in FaDu. OM = oligomycin; R = rotenone; AA = antimycin A; mRNA sub. = mRNA subtype; E_RT-DC_ = Young’s modulus, real-time deformability cytometry, E (AFM) = Young’s modulus, atomic force microscopy.

Coculture of cancer cells with patient-derived CAFs demonstrated that only some CAFs support the colony-forming of cancer cells and that this phenomenon is cancer cell type-specific (Figure 2a,b). Accordingly, based on a difference to non-cocultured cells, it is possible to create groups of cancer cell colony-supporting and -non-supporting CAFs separately for FaDu and Detroit 562 cells (Figure S2). Interestingly, the degree of cancer cell-colony forming support by CAFs is associated with the tissue-of-origin mRNA subtype (Figure 2c). For the metastatic Detroit 562 cell line, the least supportive CAFs were those derived from the classical (CL) mRNA subtype of HNSCC tumour mass. On the contrary, these CL CAFs were the most supportive of the colony-forming capacity of the primary FaDu cell line. ME CAFs showed the least support for colony-formation of FaDu cells (Figure 2c). It was also obvious, that some mRNA subtypes of CAFs such as AT and BA CAFs can support the growth of both types of tumour cells (primary tumour, metastasis), while CL CAFs supported only the colony-forming capacity of FaDu cells (Figure 2c, Table S3).

Interestingly, a similar mRNA-subtype-related association was also observed for the glycolysis levels (Figure 2d,e). The coculture of cancer cells with CL, AT, and ME CAFs significantly increased glycolysis in FaDu cells. Cancer cell glycolysis levels were in a positive correlation with proliferation (proliferation-promoting) in FaDu cells but were in negative correlation with proliferation in Detroit 562 cells (Figure 2f,g, Table S4). Gene expression of *MCT1*, *MCT4*, and *CD147* was, therefore, performed to verify whether lactate transport is responsible for this proliferation promotion. These lactate shuttle-related genes were downregulated in cancer cells by coculture with supporting CAFs (Figure 2f and Figure S3). However, the expression of lactate transporters in Detroit 562 derived from metastatic cells was significantly less influenced by coculture. The increased area of colonies was shown to be in a negative correlation with *MCTs* and *CD147* expression only in FaDu primary tumour cells, this effect was not observable in metastatic Detroit 562 cells Figure 2g. Furthermore, the cell division rate assessed by QPI was in negative correlation with *MCT4* expression in FaDu cells cocultured with CAFs (Figure 2h,i).

In summary, these data indicate the cancer cell colony-supporting effect is driven by the mRNA subtype of the tumour from which the CAFs are derived, and this degree of colony-forming support is reflected in cancer cell glycolysis level and lactate shuttle-related transporters.

### 3.4. Cell Stiffness of FaDu Cells Is Associated with Mitochondrial ATP Production and MCT1 Expression

**Figure 3:**
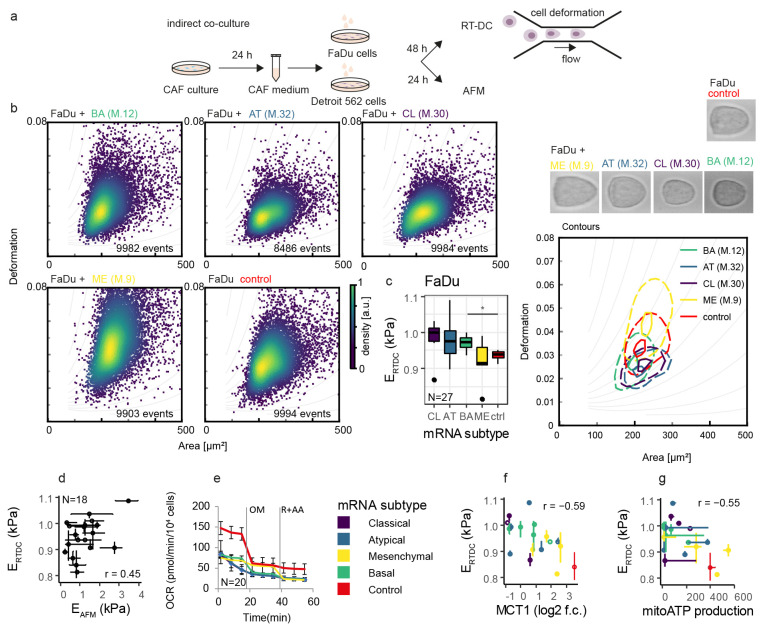
Cancer cell stiffness is affected by CAF coculture. (**a**) schematic of indirect (conditioned media-based) coculture experimental scheme used for cancer cell mechanophenotypisation. (**b**) Real-time deformability cytometry (RT-DC) scatterplots show the dependence of cell deformation on cell size. One dot represents a single cell. Representative scatterplots of FaDu cells cocultured with a CAF-conditioned medium (left) and contour plots of these scatterplots with representative cells (image width 25 µm). CAFs were derived from four tumour tissue mRNA subtypes: BA, basal; AT, atypical; CL, classical; ME, mesenchymal. For all patients tested see Figure S4. (**c**) Young’s modulus determined by RT-DC (E_RT-DC_) for four major mRNA subtypes. Performed in duplicates per CAF coculture treatment, N indicates the number of cocultures, asterisk indicates statistical significance at *p* < 0.05. (**d**) Young’s modulus determined by RT-DC and atomic force microscopy weakly correlates; for AFM measurements see Figure S4. (**e**) The oxygen consumption rate (OCR) of FaDu cells is affected by the CAF mRNA subtype and is in negative correlation with Young’s modulus (**g**), and *MCT1* FaDu expression (**f**) OM = oligomycin; R = rotenone; AA = antimycin A; E_RT-DC_ = Young modulus, real-time deformability cytometry, E_AFM_=Young modulus, atomic force microscopy.

Together with cancer cell migration, changes in the cell mechanical phenotype are important in vitro indicators of cell aggressiveness [27]. As demonstrated previously, distinct cellular mechanic properties support the malignant aggressive potential, including cell migration [28]. Measurement of cell stiffness was therefore performed after coculture. Coculture of tumour cells with CAF-derived conditioned media significantly affects the mechanical properties of cancer cells, as demonstrated by real-time deformability cytometry (Figure S4) and atomic force microscopy (Figure S5). Similarly to the effect of CAFs on colony-forming capacity and lactate shuttle, the coculture effect on cell stiffness is mRNA-subtype specific (Figure 3a,b). Coculture of FaDu cells with the BA subtype of CAFs led to an increase in stiffness of FaDu cells (see Figure 3c). Results obtained from both methods were in positive correlation (see Figure 3d). Furthermore, the value of cell stiffness was negatively associated with mitochondrial ATP production and *MCT1* expression in FaDu cells (see Figure 3e–g). Interestingly, CAFs from ME tumours cause the most pronounced decrease of cancer cell stiffness, accompanied by minimal changes in lactate shuttle, and conversely, CAFs from CL tumours cause the most pronounced stiffening accompanied by the highest decrease of MCT1.

### 3.5. Cancer Cells Can Manipulate Their CAFs

**Figure 4:**
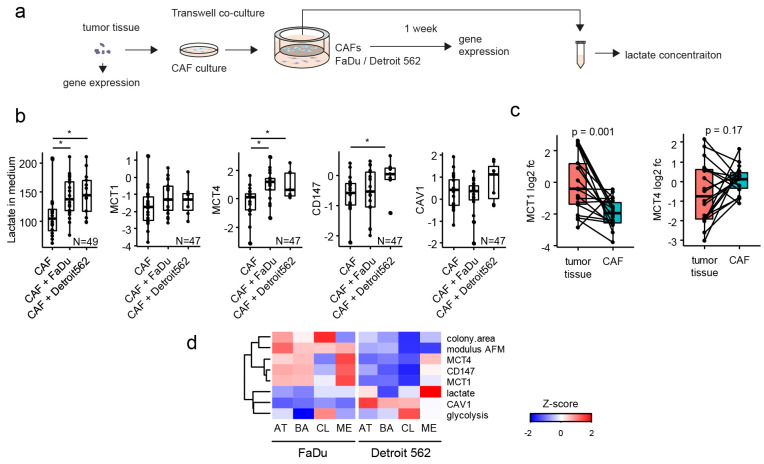
Coculture of the CAFs with cancer cell lines increases lactate in medium and lactate shuttle-relevant gene expression in CAFs. (**a**) scheme of measurement, (**b**) levels of lactate in medium and lactate-shuttle-relevant genes expression level in CAFs, asterisk indicate *p* < 0.05 in paired *t*-test. (**c**) *MCT1* and *MCT4* gene expression in tumour tissue and corresponding tumour tissue-derived CAFs. *p* indicates the result of paired *t*-test, individual dots represent individual patient-derived CAFs. (**d**) summary of the coculture data –shown as an integrative heatmap of measured data on cocultured cells, shown as a mean-per-mRNA subtype (CL, classical, AT, atypical, BA, basal, ME, mesenchymal).

The effect of coculture on the lactate transporters’ gene expression was bidirectional as the coculture of patient-derived CAF cell lines with FaDu and Detroit 562 cells also had a significant effect on the gene expression of *MCT4* and *CD147* in CAFs (see Figure 4a,b). The CAFs showed an increase in lactate production and *MCT4* expression due to coculture with cancer cells. An increase of *CD147* expression in the CAFs was specific for the coculture with Detroit 562 cells. Furthermore, *MCT1* expression in tumour tissues was significantly higher than the expression in the CAFs (see Figure 4c) but *MCT4* expression was not changed between tumour tissue and the CAFs. Therefore, high *MCT1* expression in tumour tissue can be attributed to cancer cells.

In conclusion, the data indicate a bi-directional interplay in lactate shuttling between CAFs and cancer cells (Figure 4d), where changes in lactate transporters are accompanied by an increase of extracellular lactate when cocultured.

### 3.6. Patients with Basal mRNA Subtype of HNSCC and Overexpression of Lactate Transport-Associated Genes Have a Poor Prognosis

**Figure 5:**
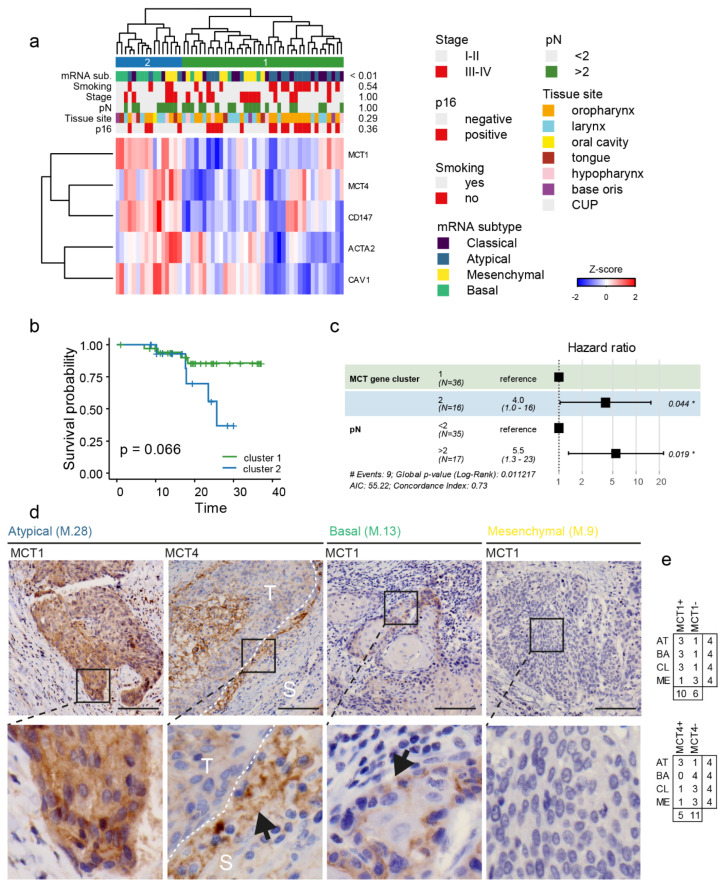
Lactate shuttle-relevant gene expression clusters in tumour tissues are associated with patient survival. (**a**) heatmap of tumour tissue lactate shuttle-relevant gene expression in 55 primary head and neck tumours. *p* values on the right side indicate Fischer’s exact test for p16 status tissue site, node status, cancer stage, smoking, and mRNA subtype. Clustering results in two clusters, which demonstrated an overall survival trend in the Kaplan-Meier chart (**b**). (**c**) Cox regression, stepwise model. Gene expression cluster is an overall survival predictor independent of tumour node status (pN), p16 status, and tumour stage, which were used as input in the Cox model. Asterisk highlights displayed *p*-values lower than 0.05. (**d**) MCT1 and MCT4 expression in the stroma and tumorous tissues, representative basal, mesenchymal, and atypical mRNA subtype tumours. MCT1 cytoplasmic positivity and isolated membrane positivity in tumour cells, of AT tumours (first column), MCT4 weak positivity in tumour tissue and strong positivity of inflammatory elements and sparse stromal cells in peritumoral stroma (S) on a periphery of tumour nests (T, indicated by the arrow, second column); MCT1 positivity of isolated tumour cells in a basal layer of the nests, BA tumours (indicated by the arrow, third column); MCT1 negativity in tumorous tissues (last column). 40×, scalebar indicates 100 μm, detail width 80 μm. (**e**) MCT1/4 tumour tissue positivity and mRNA subtype in immunohistochemical staining.

Regarding implications for a clinical picture, tissue expression of lactate transport-related genes was analysed directly in tumorous tissues of 55 patients used in the study prior to CAF isolation. The clustering based on *MCT1*, *MCT4*, *CD147*, *CAV1*, and *ACTA2* expression resulted in the formation of two clusters (Figure 5a). These clusters demonstrated an association with the mRNA subtype of tumours, with the basal subtype being predominantly located in the second cluster. Interestingly, these two clusters were not associated with any clinically relevant head and neck parameters, including tissue site, p16 status, staging, and smoking. On the other hand, an association with survival was observed: a trend, although insignificant, was observed using a Kaplan–Meier survival analysis (Figure 5b), and, more importantly, using a stepwise Cox model with tissue expression cluster, pN, staging, p16, grade, and smoking as independent predictors, it was demonstrated that “lactate gene cluster” together with tumour stage pN are independent predictors of overall survival (Figure 5c). The cluster of patients with worse overall survival was characterised by high expression of lactate-related transporters as well as high *ACTA2* and *CAV1* expression in tumorous tissues. Interestingly, when the expression of these genes was dichotomized by median (high/low) none of them were demonstrated to be an independent overall survival predictor, indicating that rather a specific interplay between all those genes is characteristic of tumour aggressiveness and hence patient outcome. Accordingly, CAFs supporting the expression of *MCT4* in Detroit 562 cells came significantly more often from patients who later developed recurrence (Table S3).

Regarding tissue MCT1 and MCT4 expression patterns, a partial agreement was observed with mRNA expression data; for MCT1, positivity was detected in the membrane of isolated cancer cells rather than as a homogeneous expression in tumour tissue. In accordance with mRNA MCT1 expression and immunohistochemical analysis, positivities were observed in tumours classified as basal (3/4 samples were positive) as well as atypical (3/4) and classical (3/4). On the other hand, tumours classified as mesenchymal were negative (1/4) for MCT1 expression (Figure 5d). The stromal expression was negative for MCT1. MCT4 followed a similar trend in tumorous tissues and demonstrated isolated positivity in the stroma (rather inflammatory elements) of some tumorous tissues (Figure 5d).

## 4. Discussion

Cancer-associated fibroblasts (CAFs) play many important roles in the progression of a tumour. They produce proteins of the extracellular matrix and secrete inflammatory and growth factors promoting cancer progression, cancer cell proliferation, and therapy resistance [2]. Many studies indicate that CAFs are necessary for HNSCC progression [5,29]. However, some works indicate that CAFs may also suppress tumour progression in some circumstances [30]. Both the tumour-promoting and tumour-suppressing effects of CAFs were supported by our coculture experiments.

CAFs are not a homogenous cellular type but display significant molecular and metabolic heterogeneity and high levels of metabolic plasticity. In this study, we investigated the interaction between different mRNA subtypes of CAFs and HNSCC cells and its impact on the key characteristics of these cancer cells. The mRNA subtypes of tumour tissues were determined according to Lawrence et al. [11]. Based on our coculture experiments, it was shown that CAFs derived from different mRNA subtypes of HNSCC tumour tissue had different abilities to promote the colony-forming capacity or modulate the mechanical properties of HNSCC cell lines of dissimilar origin, indicating the presence of divergent transcriptional programmes during disease progression. Consequently, fibroblasts in metastases can be activated in a different way than CAFs inhabiting primary tumours [31]. For the metastatic Detroit 562 cell line, the least supportive CAFs were those derived from the CL mRNA subtype of HNSCC tumour mass characteristic by αSMA-low, elevated levels of oxidative stress response genes, including *NFE2L2* and *SOX2*, and a history of heavy smoking. On the other hand, the most supportive were CAFs derived from the ME mRNA subtype of HNSCC tumour mass, characterised by high expression of EMT markers αSMA, vimentin, and desmin and low gene expression of the *TP63*. The level of support is cancer cell-type-specific; CL CAFs were the most supportive for the colony-forming capacity of the primary FaDu cells, and ME CAFs showed the least support for colony formation of FaDu cells (Figure 2c). It was also obvious that CAFs derived from the AT (αSMA-low, SOX2-high, p16+) or BA (αSMA- and EGFR-high and low SOX2/TP63 ratio) mRNA subtypes could support the growth of both types of tumour cells (primary-tumour ones as well as metastatic-derived ones), while CL and ME CAFs were more specific. The right type of cooperation between CAFs and cancer cells may be needed for tumour progression. For example, the proliferation of primary cancer cells not yet prepared for EMT can be suppressed by the ME subtype of CAFs, as was shown by our results. The CAFs inducing high expression of *MCT1* in FaDu cells did not support the proliferation of FaDu cells. MCT1 expression was associated with epithelial to mesenchymal transition (EMT) [32]. In epithelial cells, autocrine ERBB3 activation sustains PI3K signalling. After EMT, downregulation of ERBB3 interrupts autocrine signalling to PI3K, which leads to reduced proliferation after EMT [33]. The increased area of colonies was shown to be in negative correlation with MCT1 expression only in primary tumour cells, this was not true for the metastatic Detroit 562 cells carrying the activation mutation of PI3K. FaDu did not carry PI3K mutations [25] and therefore the colony-forming ability of FaDu cells can be diminished by the EMT process supported by ME CAFs. Furthermore, EMT softens head and neck cancer cells to facilitate migration in 3D environments [34], making changes in the cell mechanical phenotype, an important in vitro indicator of cell aggressiveness [27]. Indeed, coculture of FaDu cells with BA subtype of CAFs increased the cell stiffness of FaDu cells, which implicates EMT inhibition by these fibroblasts.

The differences between FaDu and Detroit 562 cells might not only be related to the primary versus metastatic status of the cells but also (partially) due to the different mutations carried by these cells. In addition to the aforementioned presence of the activating PI3K mutation in Detroit 562 cells, the Detroit 562 cell line has a p53 R175H mutation [35]. Though R175H p53 loses the function of the wild-type p53, p53-R175H aggregates can not only induce coaggregation of wild-type p53 to cause the loss of function, but also induce coaggregation of p63 and p73 to cause a gain of function. p63 and p73 mutations are rare in tumours, but their function can be disrupted by coaggregation with mutant p53, leading to an increase in the oncogenic potential of the affected cells [36]. p63 supports aerobic respiration and p63-depleted cells had a significantly reduced oxygen consumption rate [37]. Depletion of p73 caused altered lysine metabolism and glycolysis, as well as an elevated pentose phosphate pathway and abnormal lipid accumulation. p73 has been shown to regulate metabolic enzymes, such as cytochrome c oxidase subunit IV isoform 1, glucose 6-phosphate dehydrogenase, and glutaminase-2 [38]. In the FaDu cell line, a p53 mutation occurs at codon 248 (p.R248L) [39]. A codon 248 p53 mutation preserves some tumour suppressor function [40]. Homozygous deletion of SMAD4 was also observed in FaDu cells [41,42]. SMAD4 gene depletion can induce autophagy. The protein level of SMAD4 was inversely correlated with autophagy in orthotopic tumour tissue samples [43]. Furthermore, primary tumour cells (FaDu) contained 63.8% cells with CD44-negative status and only 36.1% cells with CD44+ status, 95.5% of metastatic Detroit 562 cells showed CD44+ status. CD44 triggers EMT in many cancers [44,45]. Therefore, EMT stimulating ME subtype of CAFs can probably cooperate better with CD44-positive metastatic Detroit 562 cells. Data indicates that the changes in cancer cell mechanics are linked also with their metabolic reprogramming. In the literature, evidence exists on the level of interplay between stromal stiffness and cancer cell OXPHOS [46] or glycolysis [47]. Nevertheless, the crosstalk between the cell mechanics and metabolic reprogramming is far from being clearly understood. However, coculture experiments performed in this study provide evidence that cancer cell stiffness is in negative correlation with their MCT1 expression and OXPHOS, suggesting that lactate shuttle and CAF-mediated HNSCC metabolic reprogramming are also associated with the support of aggressive potential via cell stiffness modulation [28]. Metabolic reprogramming is most likely used to sustain active processes in the actin cortex of cancer cells [48]. Similarly to colony-forming capacity, cell softening is an mRNA-subtype specific with the most profound softening after ME subtype coculture.

Interestingly, the coculture of cancer cells with CL CAFs significantly increased glycolysis in FaDu cells (Figure 2d and Figure 4d). Glycolysis was rather proliferation-promoting in FaDu cells, but was in negative correlation with proliferation in Detroit 562 cells, Figure 2g. Enhanced glycolysis can provide many important advantages helpful for cancer cells such as the possibility of rapid ATP synthesis, higher flux into biosynthetic pathways, and immune cell evasion [49]. Nevertheless, metastatic cancer cells without functioning oxidative phosphorylation (OXPHOS) show reduced proliferation, low migration and decreased metastatic potential. Mitochondrial ATP synthesis is not required to overcome these obstacles. On the other hand, the respiratory chain and dihydroorotate dehydrogenase (DHODH)-dependent pyrimidine biosynthesis is crucial [50,51].

Consequently, metastatic Detroit 562 cells can prefer higher OXPHOS and CAFs forcing them to glycolysis can be counterproductive for their progression. Furthermore, lactate promotes glutamine uptake and metabolism in oxidative cancer cells and these cancer cells can use lactate as fuel supporting their growth and division [52,53]. All subtypes of CAFs cocultured with cancer cells showed an increase in lactate production and *MCT4* expression (see Figure 4b) suggesting metabolic manipulation by cancer cells and the possible establishment of metabolic symbiosis. It was observed that fibroblasts surrounding malignant cells have a low Cav-1 expression, high MCT4 expression and enhanced aerobic glycolysis, which mirrors the simultaneous increase of mitochondrial activity in the adjacent epithelial cancer cells [54]. An increase of the *CD147* expression was specific for the coculture of CAFs with Detroit 562 cells. CD147 (extracellular matrix metalloproteinase inducer, EMMPRIN), a transmembrane glycoprotein and the chaperone of MCT1 and MCT4, has been studied as a potential prognostic marker of HNSCC. As its name suggests, CD147 has been thought to act mainly as an inducer of MMPs; however, its role in facilitating the cell membrane localization and functionality of MCTs implicates a dominant role in the regulation of aerobic glycolysis. It has been reported that CD147 plays a role in the transformation of normal fibroblasts into CAFs through the induction of α-SMA expression [55].

Regarding implications for the clinical picture, a group of patients with overexpression of lactate transport-associated genes had poor overall survival rates. This group contained most of the patients with basal mRNA subtype of HNSCC. CAFs supporting the expression of *MCT4* in Detroit 562 cells came significantly more often from patients who later developed a recurrence. The BA subtype is characterised by high expression of *EGFR*, which can signalize CD44-positivity as CD44 upregulates the expression of EGFR, leading to the activation of the PI3K/Akt signalling pathway [45]. The BA subtype was αSMA-high which suggests the presence of myofibroblastic CAFs. Across various cancer types, myofibroblastic CAFs were associated with ECM production [30,56]. In HNSCC, the presence of myofibroblastic CAFs upregulated the expression of galectin-1 [57]. Galectin-1 in ECM modifies the effect of ECM on cells by influencing cell adhesion, cell migration through ECM, and cell death [58]. Targeting galectin-1 production in CAFs inhibited HNSCC metastasis [59]. Furthermore, the BA subtype of CAFs was able to support the growth of both primary tumour cells (FaDu) and metastatic cells (Detroit 562).

## 5. Conclusions

Cancer-associated fibroblasts play many important roles in the progression of HNSCC. Different cellular origins and factors produced by tumour cells shape the phenotype of CAFs and contribute to their heterogeneity and the presence of distinct CAF subtypes. The identity and proportion of these subtypes could differ across normal, premalignant, and malignant states, mutation statuses, and also between metastatic and primary tumours. Based on our results, we assume that the right kind of cooperation between cancer cells and CAFs is needed and premature support of some steps of cancerogenesis, such as EMT, can suppress cancer cells in some circumstances. A better insight into the CAF subpopulations emerging during cancer progression is crucial for successful therapeutic targeting of symbiosis between CAFs and cancer cells. As various CAF subpopulations have distinct roles in tumour progression, targeting them individually may lead to better outcomes. A deep characterization of CAF subtypes will therefore be pivotal for the design of effective combinatorial targeting of CAFs and cancer cells.

## Figures and Tables

**Table 1:** Clinico-pathological characteristics of 55 patient cohort. Shown as number (% per group). *p*-values for Fischer exact test for factor vs. mRNA subtype and for a lactate shuttle-relevant gene expression clusters (see further in Figure 5), respectively. AT, atypical, BA, basal, CL, classical, ME, mesenchymal. N, number of patients.

Factor/Level	*N*	mRNA Subtype				Lactate Shuttle Gene Cluster	
		AT	BA	CL	ME	1	2
Gender, *p*-value = 0.974/0.519							
F	14	4 (28.6%)	3 (21.4%)	4 (28.6%)	3 (21.4%)	9 (64.3%)	5 (35.7%)
M	41	14 (34.1%)	8 (19.5%)	10 (24.4%)	9 (22.0%)	30 (73.2%)	11 (26.8%)
Tumor location, *p*-value < 0.001/0.286							
floor of the mouth	3	0 (0.0%)	1 (33.3%)	1 (33.3%)	1 (33.3%)	2 (66.7%)	1 (33.3%)
hypopharynx	6	1 (16.7%)	0 (0.0%)	3 (50.0%)	2 (33.3%)	4 (66.7%)	2 (33.3%)
larynx	16	0 (0.0%)	5 (31.2%)	9 (56.2%)	2 (12.5%)	12 (75.0%)	4 (25.0%)
oral cavity	1	0 (0.0%)	1 (100.0%)	0 (0.0%)	0 (0.0%)	0 (0.0%)	1 (100.0%)
oropharynx	24	17 (70.8%)	2 (8.3%)	1 (4.2%)	4 (16.7%)	19 (79.2%)	5 (20.8%)
tongue	5	0 (0.0%)	2 (40.0%)	0 (0.0%)	3 (60.0%)	2 (40.0%)	3 (60.0%)
p16 status, *p* < 0.001/0.3594							
p16 neg.	35	0 (0.0%)	10 (28.6%)	14 (40.0%)	11 (31.4%)	23 (65.7%)	12 (34.3%)
p16 pos.	20	18 (90.0%)	1 (5.0%)	0 (0.0%)	1 (5.0%)	16 (80.0%)	4 (20.0%)
pN, *p*-value = 0.03059/1.000							
<2	37	12 (32.4%)	9 (24.3%)	12 (32.4%)	4 (10.8%)	26 (70.3%)	11 (29.7%)
>2	18	6 (33.3%)	2 (11.1%)	2 (11.1%)	8 (44.4%)	13 (72.2%)	5 (27.8%)
Stage, *p* value < 0.001/1.000							
I–II	28	16 (57.1%)	5 (17.9%)	3 (10.7%)	4 (14.3%)	20 (71.4%)	8 (28.6%)
III–IV	27	2 (7.4%)	6 (22.2%)	11 (40.7%)	8 (29.6%)	19 (70.4%)	8 (29.6%)
Smoking status, *p* < 0.001/0.536							
0	18	14 (77.8%)	0 (0.0%)	0 (0.0%)	4 (22.2%)	14 (77.8%)	4 (22.2%)
1	37	4 (10.8%)	11 (29.7%)	14 (37.8%)	8 (21.6%)	25 (67.6%)	12 (32.4%)

## Data Availability

The data that support the findings of this study are available from the corresponding author upon request.

## Supplementary Materials

The following supporting information can be downloaded at: https://www.mdpi.com/article/10.3390/cancers14092286/s1, Table S1: Clinical characteristics of the patient cohort; Table S2: Clinical characteristics of a subset of the patient cohort used for CAF generation for coculture experiments; Table S3: Cancer cells cocultured with HNSCC-derived cancer-associated fibroblasts, descriptive statistics and results of ANOVA; Table S4: Cancer cells cocultured with HNSCC-derived cancer-associated fibroblasts, results of Pearson correlation; Table S5: Summary of clinico-pathological characteristics of patients from which CAF cultures were established. All patients were indicated for surgery with a curative intent. The table is supplemented by the mRNA subtype; Table S6: Raw data used for analysis; Figure S1 Lineage specificity of cancer-associated fibroblasts, CD90/SYTOXblue status determined by flow cytometry; Figure S2: Colony area of FaDu and Detroit 562 cells cocultured in Transwell with cancer-associated fibroblasts; Figure S3: *MCT1*, *MCT4*, *CD147*, and *CAV1* gene expression in FaDu and Detroit 562 cells cocultured with cancer-associated fibroblasts; Figure S4: FaDu cancer cell stiffness is affected by coculture with head and neck-derived cancer-associated fibroblasts; Figure S5. FaDu and Detroit 562 cancer cell stiffness is affected by coculture with head and neck-derived cancer-associated fibroblasts.

## Abbreviations

ACTA2	actin alpha 2
AFM	atomic force microscopy
AT	atypical mRNA subtype
BA	basal mRNA subtype
CAF	cancer-associated fibroblast
CAV	caveolin
CD147	cluster of differentiation 147
CL	classical mRNA subtype
DHODH	dihydroorotate dehydrogenase
ECAR	extracellular acidification rate
EMT	epithelial-mesenchymal transition
FFPE	formalin-fixed, paraffin-embedded
HNSCC	head and neck squamous cell carcinomas
HPV	human papilloma virus
MCT	monocarboxylate transporter
ME	mesenchymal mRNA subtype
OCR	oxygen consumption rate
OXPHOS	oxidative phosphorylation
RT-DC	real-time deformability cytometry
TME	tumour microenvironment
VIM	vimentin
