# Supplementary material for: mRNA Subtype of Cancer-Associated Fibroblasts Significantly Affects Key Characteristics of Head and Neck Cancer Cells
Source: Cancers (Basel). 2022 May 3;14(9):2286. doi: 10.3390/cancers14092286 (PMC9102192; doi:10.3390/cancers14092286)
Supplement: Supplementary file 1 [file cancers-14-02286-s001.zip › Supplementary information.pdf]

Supplementary information

mRNA subtype of cancer-associated fibroblasts significantly affects key characteristics of head and neck cancer cells

Barbora Peltanová[1,2], Hana Holcová Polanská[1,2], Martina Raudenská[1,2,3], Jan Balvan[1,2], Jiří Navrátil[1], Tomáš Vičar[2], Jaromír Gumulec[1], Barbora Čechova[1], Martin Kräter[5], Jochen Guck[4], David Kalfeřt[5], Marek Grega[6], Jan Plzák[5], Jan Betka[5], Michal Masařík[1,2,7,*]

[1]Department of Pathological Physiology, Faculty of Medicine, Masaryk University/Kamenice 5, CZ-625 00 Brno, Czech Republic; bpeltanova@seznam.cz (B.P.); j.gumulec@med.muni.cz (J.G.); m.raudenska@gmail.com (M.R.); hana.polanska@gmail.com (H.H.P.)

[2]Department of Physiology, Faculty of Medicine, Masaryk University/Kamenice 5, 62500 Brno, Czech Republic

[3]Department of Chemistry and Biochemistry, Mendel University in Brno, Zemedelska 1, 61300 Brno, Czech Republic

[4] Max Planck Institute for the Science of Light, Staudtstraße 2, 91058 Erlangen, Germany

[5]Department of Otorhinolaryngology and Head and Neck Surgery, University Hospital Motol, First Faculty of Medicine, Charles University, V Uvalu 84, 15006 Prague 5, Czech Republic; david.kalfert@fnmotol.cz (D.K.); jan.plzak@lf1.cuni.cz (J.P.); jan.betka@fnmotol.cz (J.B.)

[6]Department of Pathology and Molecular Medicine, 2nd Faculty of Medicine, Charles University and University Hospital Motol, V Uvalu 84, 15006 Prague 5, Czech Republic; marek.grega@fnmotol.cz

[7]BIOCEV, First Faculty of Medicine, Charles University, Prumyslova 595, 25250 Vestec, Czech Republic

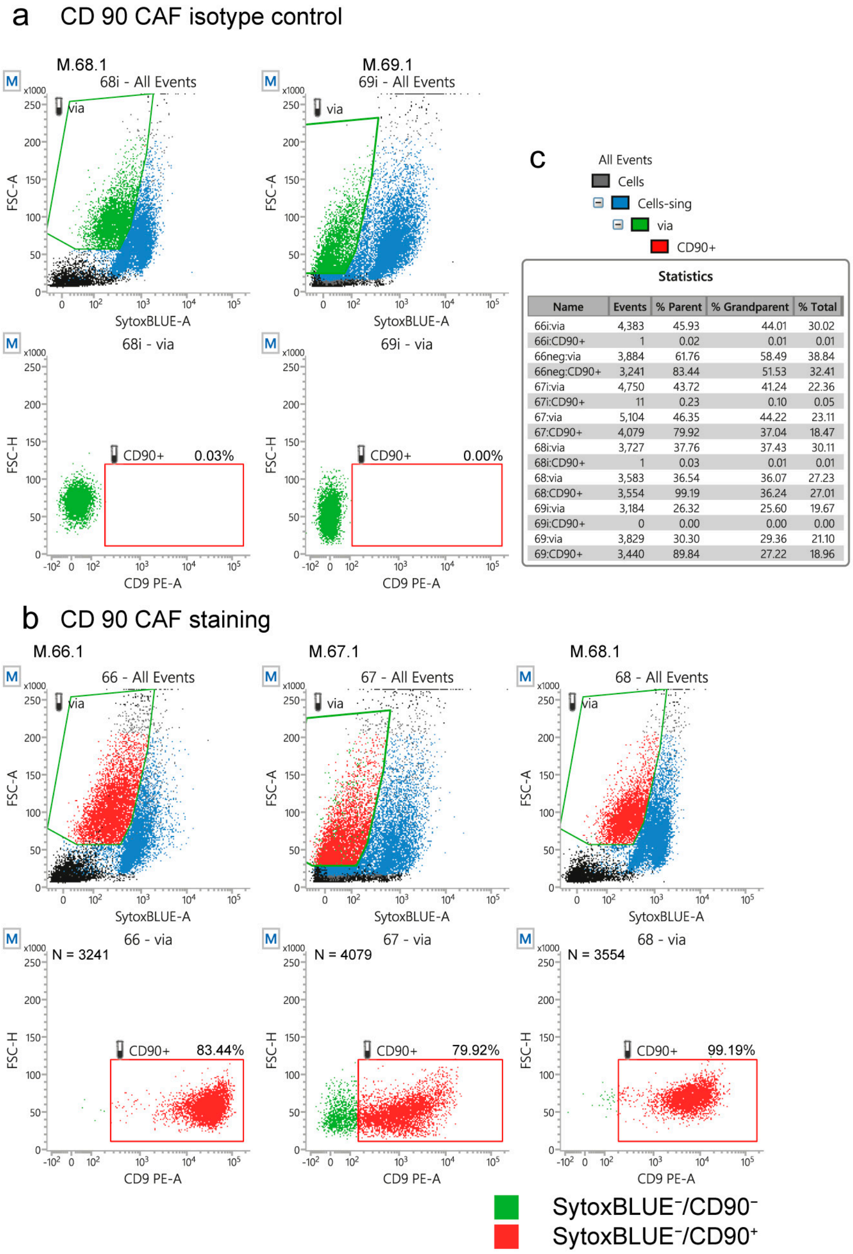

Name	Events	% Parent	% Grandparent	% Total
66i:via	4,383	45.93	44.01	30.02
66i:CD90+	1	0.02	0.01	0.01
66neg:via	3,884	61.76	58.49	38.84
66neg:CD90+	3,241	83.44	51.53	32.41
67i:via	4,750	43.72	41.24	22.36
67i:CD90+	11	0.23	0.10	0.05
67:via	5,104	46.35	44.22	23.11
67:CD90+	4,079	79.92	37.04	18.47
68i:via	3,727	37.76	37.43	30.11
68i:CD90+	1	0.03	0.01	0.01
68:via	3,583	36.54	36.07	27.23
68:CD90+	3,554	99.19	36.24	27.01
69i:via	3,184	26.32	25.60	19.67
69i:CD90+	0	0.00	0.00	0.00
69:via	3,829	30.30	29.36	21.10
69:CD90+	3,440	89.84	27.22	18.96

Figure S1 Lineage specificity of Cancer-associated fibroblasts, CD90/SYTOXblue status determined by flow cytometry. Positivity of CD90 status tested on viable SYTOXblue-negative population. **a.** isotype control for tested samples showing negativity for CD90, **b.** CD90/SYTOXblue staining for representative CAF populations. **c.** gating hierarchy and table with descriptive statistics for tested CAF samples. note “i” indicates isotype control.

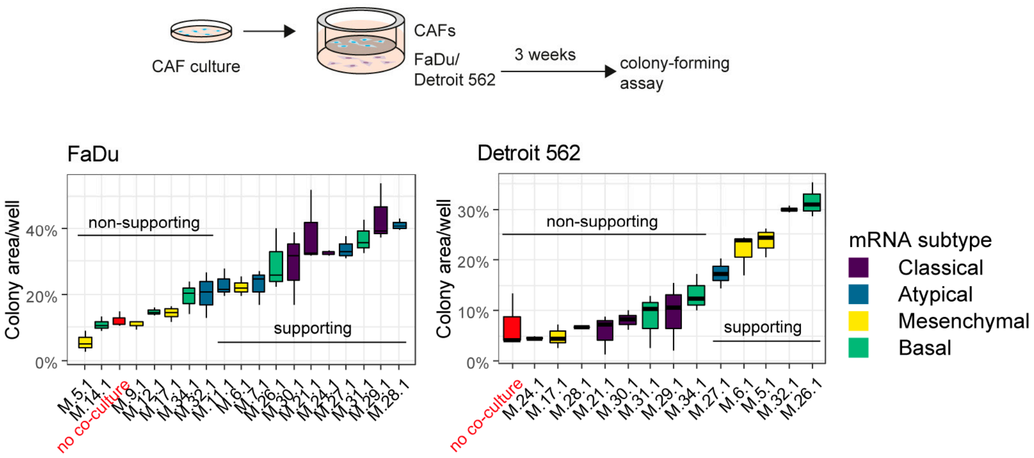

Figure S2: Colony area of FaDu and Detroit cells co-cultured in Transwell with cancer-associated fibroblasts. schematic of the experiment (top) and measurement of colony area per well area relative to non-co-cultured FaDu and Detroit 562 cancer cells. The supporting status is defined by $p < 0.05$ compared to non-cocultured cells or >2fold increase in colony area.

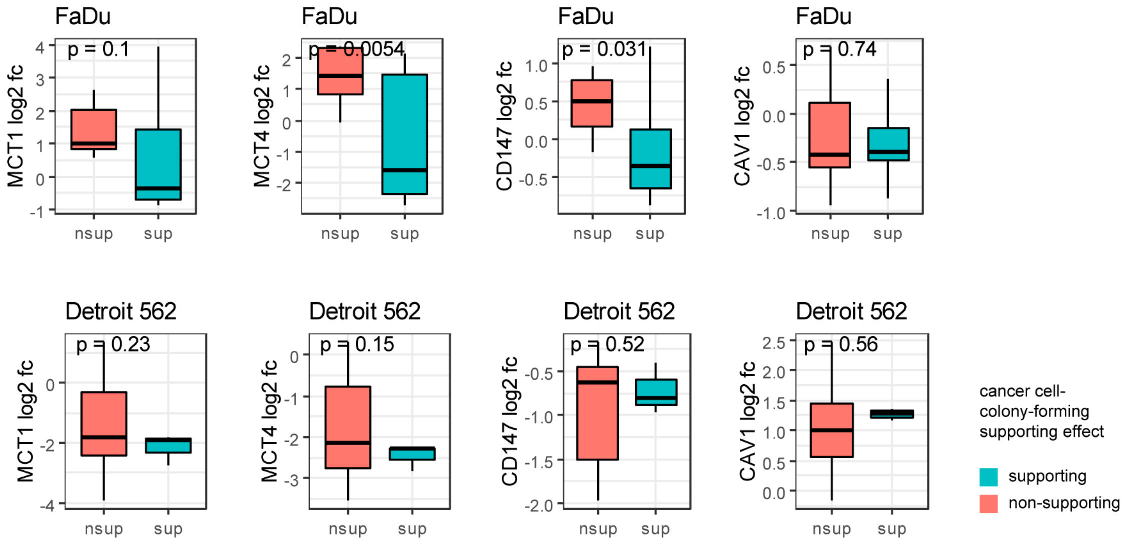

Figure S3: MCT1, MC4, CD147 and CAV1 gene expression in FaDu and Detroit 562 cells co-cultured with cancer-associated fibroblasts, t-test by CAFs' colony-supporting status.

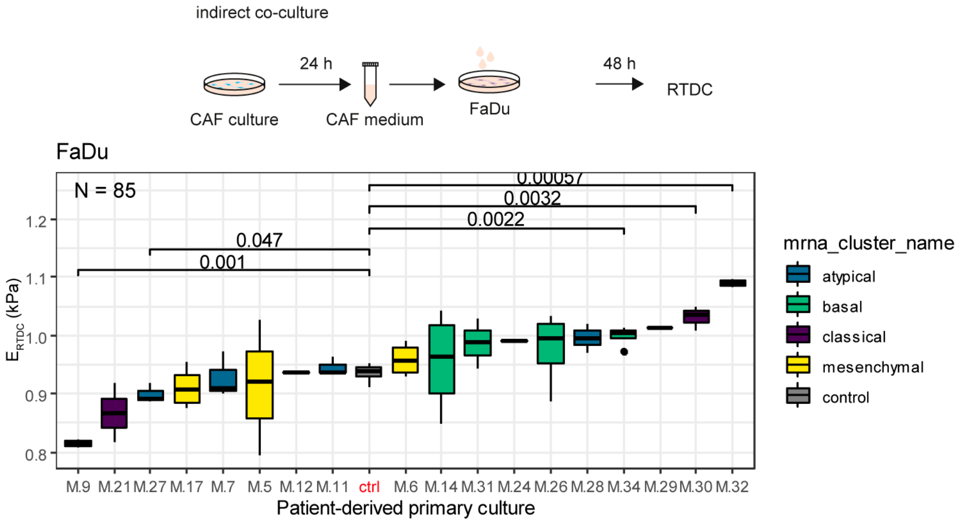

Figure S4: Cell stiffness of FaDu cancer cells is affected by co-culture with head and neck-derived cancer-associated fibroblasts. schematic of the measurement (up) and Young modulus of FaDu determined by real-time deformability cytometry (RTDC). Every measurement is one of the at least duplicate measurements. p-values of BH-adjusted t-test relative to stiffness of FaDu cells in depleted medium.

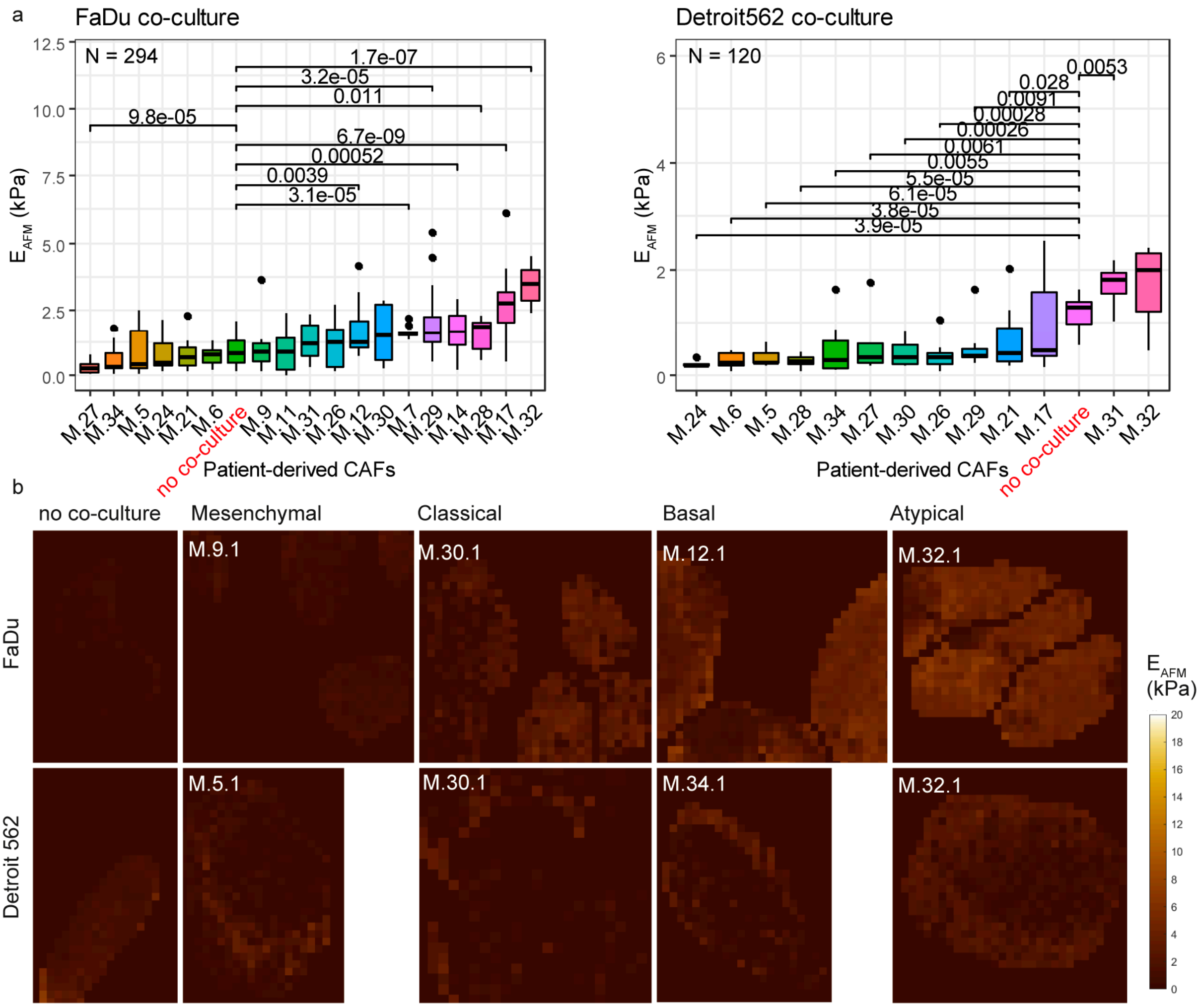

Figure S5. **Cell stiffness of FaDu and Detroit 562 cancer cells is affected by co-culture with head and neck-derived cancer-associated fibroblasts**. Young's modulus of FaDu and Detroit 562 determined by atomic force microscopy. Eight FOVs per CAF co-culture were typically used. p-values of BH-adjusted t-test relative to stiffness of non-cocultured cells in depleted medium.
